# Male Glow‐Worms Combine Flying With Walking and Impede One Another in Their Scramble to Reach Females

**DOI:** 10.1002/ece3.72187

**Published:** 2025-09-20

**Authors:** Estelle M. Moubarak, Alan J. A. Stewart, Jeremy E. Niven

**Affiliations:** ^1^ School of Life Sciences University of Sussex Brighton UK

**Keywords:** approach behaviour, glow‐worms, Lampyridae, male–male interactions, mate attraction, phototaxis, scramble competition

## Abstract

For male glow‐worms to achieve a mating, they must detect the bioluminescent glow emitted by females at night and subsequently reach them. Several aspects of male behaviour suggest they engage in scramble competition, with many males striving simultaneously to reach a female first and acquire a mating opportunity. Although male glow‐worms fly during the initial stages of their search, little is known about the final stages of their approach, despite this potentially involving the most intense competition. In a laboratory arena, males combine walking with flying short distances to reach a dummy female (DF; green LED), though walking predominates. To determine if this was representative of behaviour in the field, we used infrared videography coupled with DF traps. Most males landed near a DF, thereafter walking or making short bounding flights to reach it. The time taken by males to reach the DF after their initial landing increased with increasing numbers of males in the vicinity. Combining a DF attached to a wire stand with infrared videography showed that during the final approach, males engage in frequent, typically brief interactions. Greater numbers of males in the vicinity reduced the rate at which they reached the DF. After reaching the DF, males frequently fell in clusters, ending their mating opportunity. Our results show that the final stage of the males' approach to a DF (and we infer to females) is dominated by walking and influenced by interactions with other males, consistent with features of scramble competition found in other polygynous insects, including fireflies. Our findings offer novel insights into the life history of male glow‐worms and, more generally, features of scramble competition in insects.

## Graphical abstract

Using an observational approach in field and laboratory settings we investigated male glow‐worms' approach to females to determine whether their behaviour is consistent with scramble competition. Our results show that males combine flying and walking, favouring the latter during the final stage of their approach to females. Our data also show that males frequently interact with one another, reducing the rate at which they reach the female.

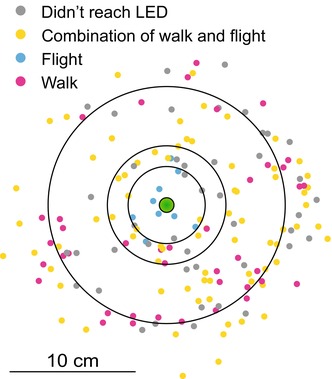

## 1 Introduction

Male mating success in many species depends on securing access to a female as quickly as possible with the lowest possible energy expenditure. In such mating systems, male success is limited by the number of females (limiting sex) with which they can mate; males invest more in female acquisition and produce low‐cost gametes, whereas females invest more in gametes (Bateman 1948). Male behaviour can differ considerably depending upon female behaviour (Emlen and Oring 1977; Thornhill and Alcock 1983). In species where females, or the resources they value, cannot be defended by males, a mating system known as scramble competition can evolve in which males strive against one another to acquire a female first (Wells 1977; Thornhill and Alcock 1983). Several features of mating systems promote scramble competition, including females being dispersed spatially and/or temporally and difficult to defend and available for mating only within a discrete period (Emlen and Oring 1977; Thornhill and Alcock 1983).

Scramble competition is common in terrestrial insects, occurring in numerous taxa. In a review of scramble competition polygyny in terrestrial arthropods, Herberstein et al. (2017) identified taxa engaging in this type of mating system from many major insect orders including Odonata, Orthoptera, Hemiptera, Coleoptera, Lepidoptera, and Diptera. The form of scramble competition, prolonged searching polygyny or explosive breeding assemblage, engaged in by an insect species depends upon female dispersal whereby solitary, highly dispersed females promote the former and clustered females promote the latter (Emlen and Oring 1977; Thornhill and Alcock 1983).

In insects engaging in scramble competition, males display traits that promote the rapid detection and acquisition of females. These traits include motor adaptations that promote rapid movement towards females, as is found in the Cook Strait giant weta (*Deinacrida rugosa*), in which males with smaller bodies and longer legs are more mobile and more successful in terms of female inseminations (Kelly et al. 2008). In some species, sexual dimorphisms may be extreme, with males being highly mobile and possessing wings, whereas females are sedentary (reviewed in Herberstein et al. 2017). Scramble competition may also promote protandry, the early emergence of males in comparison to females (Wiklund and Fagerström 1982). This is found in many insect species, such as Dawson's burrowing bee ( *Amegilla dawsoni* ), in which smaller males emerge before their larger counterparts, which may enable them to obtain copulations with females (Alcock 1997). Males also possess sensory adaptations that promote rapid detection of females, particularly the signals females of some species use to advertise their location. For example, males of the common tea tree stick insect (*Clitarchus hookeri*) are attracted to females through airborne chemical cues, and their antennae possess greater numbers of basiconic sensilla for detecting these signals than those of females (Myers et al. 2015). Thus, the traits displayed by males of species engaged in scramble competition may promote finding females rapidly and ahead of their strongest competitors, other males of their species.

Yet scrambles are often multi‐stage processes involving different behaviours at different stages. For example, males of the firefly *Photinus pyralis* are flighted but their final approach to females is made on foot (Vencl and Carlson 1998; Vencl 2004). Males of this species exhibit hyperallometric scaling of wing length relative to body size, suggesting strong selective pressure on an aspect of flight, such as the ability to search for females and cover greater distances. Yet once on the ground, larger size may be a disadvantage because males engage in aggregations in which they likely impede one another's progress (Vencl and Carlson 1998). In simulated scrambles, smaller males of *Photinus pyralis* are more likely to reach females. This points to intense competition in the final stages of the scramble that may differ from earlier stages and influence which males actually make it to a particular female first.

Scramble competition has been described through numerous studies in fireflies (reviewed in Lewis and Cratsley 2008). However, remarkably little is known about scramble competition in glow‐worms, which are also lampyrid beetles. Numerous features of glow‐worm ( *Lampyris noctiluca* ) life history suggest that their mating system involves scramble competition. There is extreme sexual dimorphism in glow‐worms; the sedentary females are larviform with large abdomens full of eggs, whereas males are smaller, flighted and possess long wings. Females are the limiting sex, and adult glow‐worms of both sexes engage in capital breeding (Tyler 2002; Hopkins et al. 2021), investing the energy reserves accumulated during their larval stages to fuel their reproduction as adults (Baudry et al. 2021; Hopkins et al. 2021), so that rapidly attaining a mate may be an essential part of their reproductive fitness. Females are sparsely dispersed across the landscape in both space and time, advertising to males using their bioluminescence. Once mated, females cease glowing, making the period they are available for mating brief (often a single night), and mating delays are associated with an increased risk of reproductive failure, stressing the need to mate rapidly (Hopkins et al. 2021). Moreover, females' dispersion also means that males cannot defend them. Searching males can substantially outnumber glowing females on any given night of the breeding season (AJAS and JEN, unpublished observation), producing a highly skewed operational sex ratio (Kvarnemo and Ahnesjo 1996). They live for many days and so can potentially mate with numerous females during a single season (AJAS and JEN, unpublished observation). Thus, glow‐worms possess morphological, physiological and behavioural features that are consistent with scramble competition in the form of prolonged searching polygyny (Emlen and Oring 1977; Thornhill and Alcock 1983; Herberstein et al. 2017).

Yet it remains unclear how such a scramble occurs. One description by Tyler (2002) suggests that males land upon glowing females circumventing a scramble on the ground and the intense competition it may involve. If this were the case, the male scramble may be far simpler than that described for other lampyrid beetles such as the firefly *Photinus pyralis* (Vencl and Carlson 1998) or may involve intense competition localised entirely on the female. Indeed, this second possibility is supported by descriptions of males levering one another off the female during mating (Tyler 2002). To determine whether the specific features of the final male glow‐worm approach to glowing females involve direct landing on the female or a period of walking towards the female after landing, we adopted an observational approach using infrared videography of a dummy female (green LED) both when males are in isolation in the laboratory and in the field. We hypothesised that were males to walk during some part of the final stages, they may be subject to interactions with other males that slow them or even prevent them from reaching the female. To test this we resolved to quantify and analyse several aspects of the final stage of males' approach: (i) the mode of movement (walking vs. flying); (ii) the time of their landing; (iii) the distance from the dummy female at which they landed; (iv) the number of other males present when they landed; (v) the number and duration of interactions among males; and (vi) how the number of males present influenced the number actually reaching the dummy female. Finally, irrespective of the mode of movement, we hypothesised that male–male interactions occur on the dummy female and influence the time males spend there. Our findings suggest that males are engaged in a scramble competition in the final stages of their approach. We discuss these in the context of studies of scramble competition in other insects.

## 2 Methods

### 2.1 Animals and Field Site

Male glow‐worms ( *Lampyris noctiluca* Linnaeus, 1767) were studied at or collected from the Mount Caburn National Nature Reserve, East Sussex (UK).

### 2.2 Capture, Maintenance and Identification of Males

To conduct experiments in the laboratory arena (see below), males were captured using a custom‐made trap with a narrowband green 555 nm LED (SSL‐LX5093PGD, Lumex Inc., Carol Stream, IL, USA) mounted approximately 20 cm from the ground (e.g., Stewart et al. 2020). The LED was powered by three 1.5 V batteries. The green LED closely resembles the ~546 nm narrowband emission of the female glow and reliably attracts male glow‐worms in both field (Ineichen and Rüttimann 2012; Bird and Parker 2014; Stewart et al. 2020; Van den Broeck et al. 2021; Kivelä et al. 2023) and laboratory experiments (Moubarak et al. 2023). After capture, males were brought into the laboratory where they were marked with acrylic paint dots on the elytra for subsequent individual identification and placed in clear plastic containers (Perspex boxes L22 × W13 × H14 or L17 × W11 × H5) in a dark room with a controlled day/night cycle of 10:14 h dark:light (dark from 13:00 to 23:00). Illumination was produced with cool white LED strips (ATOM LED lighting, Telford, UK) plugged into programmable timer switches. The plastic containers contained holes in the lids to permit air circulation and were lined with wet paper towel to maintain a high humidity. A small shelter of black cardboard allowed the males to shelter from the light. They were kept in these conditions for at least 48 h prior to being introduced into the arena.

### 2.3 Arena Video‐Recordings and Behavioural Classification

**FIGURE 1:**
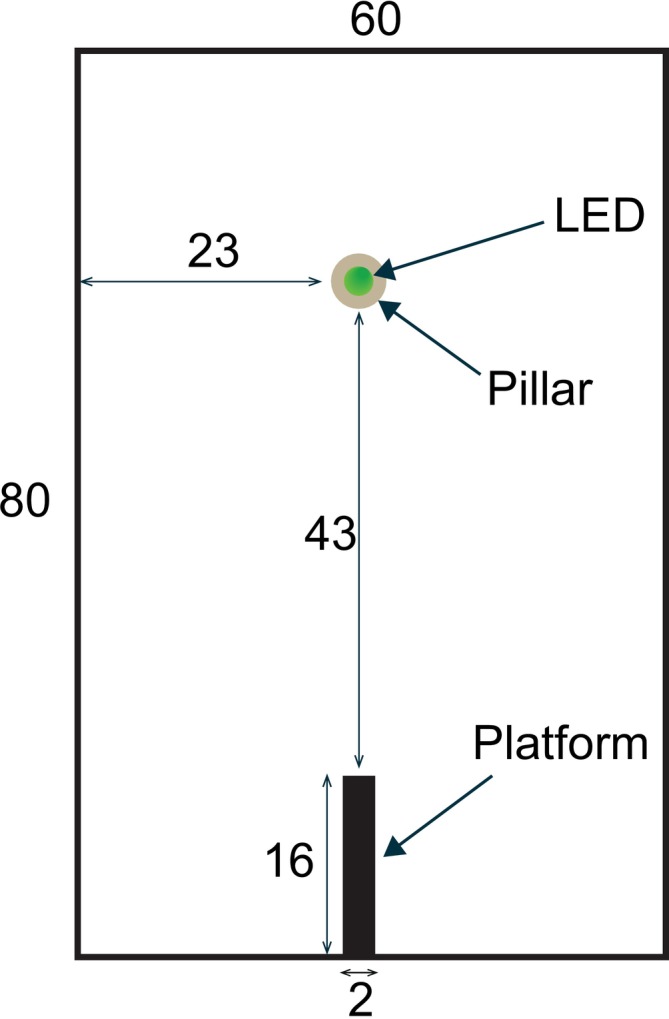
A schematic of the experimental arena. The arena is displayed from above. All measurements are given in centimetres.

To assess males' preferred means of travelling towards a female in greater detail, we constructed a rectangular arena (L80 × W60 × H60 cm) containing a platform (L16*W2) raised 4 or 9 cm above the ground. These heights were intended to correspond to those at which we have observed males climbing on grass stems at our field site prior to initiating search flights (AJAS and JEN, unpublished observation). A single green LED (as above) raised on a pillar 3 cm above the ground and 43 cm from the platform to mimic female glow (Figure 1). At the start of each experiment, an individual male was placed on the platform, and the dummy female/LED was turned on immediately. All experiments were performed in dim light conditions, and videos were recorded under infrared light using a USB infrared camera (ELP‐USBFHD05MT‐RL36‐U, Shenzhen Ailipu Technology Co., China). Recordings ended when the male reached the LED or if more than 15 min had elapsed since the male initiated a movement. Data from both platform heights were grouped because no discernible difference in the glow‐worms' behaviour could be detected.

We analysed males' mode of travel by assessing the behaviours they performed to dismount from the platform and then to travel towards the dummy female. Behaviours were classified into: ‘Leap’, males that opened their wings upon dismounting the platform but covered less than 30% of the distance between the platform and LED (13 cm); ‘Fly’, males that opened their wings upon dismounting the platform and flew more than 30% of the distance; or ‘Climb‐down’, males that climbed down the platform to the arena floor. A further behavioural classification, ‘Walk’, described males that walked with wings closed between the platform and LED. The final location of males was classified as ‘LED’ if they reached the pillar and climbed up to the LED or ‘Pillar’ if they reached within in 2 cm of the LED (Figure 4a) but did not climb. Twenty‐eight males were tested. Males that remained on the platform (*N* = 5) or fell from it (*N* = 1) were excluded from further analysis.

Videos of individual trials were analysed offline using the Fiji/ImageJ software (Schindelin et al. 2012). For the males retained for analysis (*N* = 22), we calculated approach time from the initiation of the first behaviour to the moment the male reached the pillar. The time taken to climb the LED was calculated from the moment the male reached the pillar to when it contacted the LED. Time measures are given in seconds as (mean ± standard error). Distances and speed were measured as a straight line between the locations of the male at the start and end of each behaviour.

### 2.4 Custom‐Made Infrared Video Camera

**FIGURE 2:**
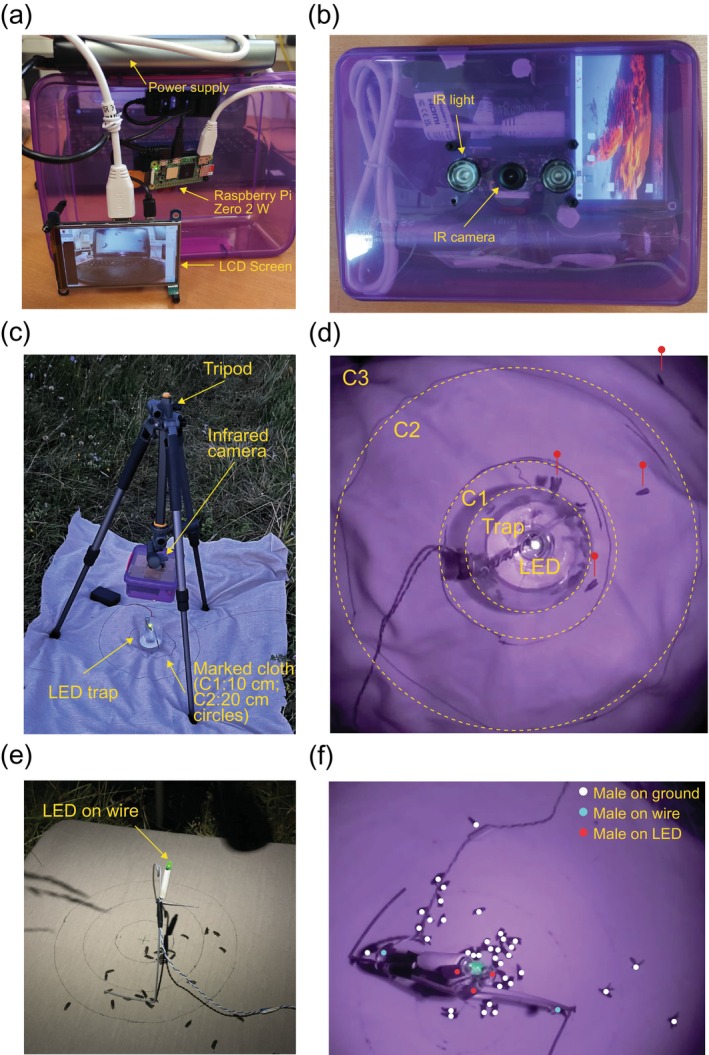
Infrared camera construction and field setup. (a) Photograph of the electrical components and wiring of the infrared camera. A Raspberry Pi single‐board computer was connected to a power supply, an LCD screen, and an infrared camera module (depicted in photograph (b)). (b) All components were fitted in a sealed transparent plastic box before being mounted on a tripod. (c) Photograph of the infrared camera setup placed above the dummy female trap in the field. The sealed box is mounted face‐down on a tripod above a single green LED (dummy female) attached to a trap. A beige cloth with circles delimiting 10 (C1) and 20 (C2) cm diameter around the LED was placed beneath the trap (see Section 2). (d) A still taken from a video recording showing the LED, the trap and the measured areas (C1, C2 and C3; see Section 2). Red markers indicate the location of three male glow‐worms on the ground and another on the rim of the trap. (e) Photograph of the dummy female attached to a wire mount in the field (camera removed from the tripod for clarity). A beige cloth with circles delimiting 5‐, 10‐ and 20‐cm diameter around the dummy female was placed beneath the wire mount (see Section 2). (f) Part of a still taken from a video recording showing the dummy female. Markers indicate the males counted on the ground, the wire and the dummy female (see Section 2).

Videos (30 frames per second) of males landing in the field were obtained using a Raspberry Pi Zero 2 W (Raspberry Pi Ltd., Cambridge, UK) connected to a night vision infrared camera module with a wide‐angle lens (160°) (Pimoroni Ltd., Sheffield, UK), a power bank (PB‐HOUMI‐1W; Elefull, Shenzhen, China), and a screen (4 in.‐HDMI‐LCD; Waveshare, Shenzhen, China) (Figure 2a,b). All electronics were sealed in a plastic container (Perspex box; L20 × W15 × H8) along with silica gel desiccant beads to keep electrical components dry (Figure 2b). The camera container was mounted on a tripod (Alta Pro 2+, Vanguard, Dorset, UK), with the lens sitting approximately 36 cm above the ground (Figure 2c). There was approximately 24 cm between the camera and LED in both configurations (see below). Once recorded, videos were opened using Fiji/ImageJ (Schindelin et al. 2012) and the ‘VideoImportExport’ plugin (https://sites.imagej.net/VideoImportExport/) for offline analysis.

### 2.5 Field Video‐Recordings and Analysis of Males Arriving at LED Traps

We used a custom‐made LED trap consisting of a green LED (see above for details; Figure 2c) mounted to face upward at approximately 12 cm from the ground above a funnel trap tapering from 8 to 2 cm (Moubarak et al. 2023). A beige cotton sheet was placed beneath the trap to enable detection of the males landing on the ground. The sheet was marked with two concentric circles of 10 and 20 cm diameter around the LED (Figure 2c,d) to permit estimation of landing distance. We recorded five one‐hour‐long videos on five separate nights, all starting at approximately 21:50 and ending at 22:50. A total of 170 males were observed across all videos. Each male's landing location (landing distance, cm) was marked and classified according to the area in which they first landed (directly in the trap, on the trap rim, or on the ground within either 5, 10 or > 10 cm of the LED; Figure 2c,d), and the landing time was recorded (landing time, minutes after sunset). Males were then tracked until they either entered the trap or exited the field of view. The time at which they entered the trap was recorded (time in trap, minutes after sunset). By subtracting the landing time from the time in trap, we estimated the time taken by each individual male to reach the LED after landing (time to LED, min). We also scored whether males that landed on the ground flew into the trap or climbed up the side of the trap to reach the LED (dummy female) and how many males walked on the LED. To assess the effect of the presence of other males on male behaviour, we counted the number of other males currently on the cloth each time a new male landed (number of neighbours). On rare occasions, video blur or small obstructions prevented us from measuring the distance or timing of males landing (*N* = 4); those males were used for all count data but not for the models where only complete observations of distance and time were retained.

### 2.6 Field Video‐Recordings and Analysis of Males Arriving at a Mounted LED

We attached a green LED (see above for details) at an upward angle of 45° at approximately 12 cm from the ground at the apex of a wire bent to resemble a parabola (Figure 2e). This configuration allows males to move freely around the dummy female (LED) instead of being removed once they reach it, and the wire ensures male behaviour is visible in the field of view. A beige cotton sheet was placed beneath the LED/wire to enable detection of males landing on the ground. The sheet was marked with three concentric circles of 5‐, 10‐ and 20‐cm diameter around the LED (Figure 2e,f).

We recorded five ~30–37‐min‐long videos on five separate nights, all starting between approximately 22:00 and 22:30. In each video, the total number of males that landed in the field of view (number of males landing) and the number of males who then reached the LED (number of males reaching LED) were counted. To estimate the proximity between males after they had landed, we selected 16 frames at random at approximately 2‐min intervals after the first landing. Individual males located on the ground, the wire or the LED were counted, and their location was recorded using the ImageJ Multi‐point tool (Figure 2f). In each frame, we extracted the Euclidean distance between each pair of males on the ground (distance between pairs of males, cm), the median distance between one male and all the other males on the cloth (median distance per male, cm), the number of males present on the ground (number of males on ground) and the number of direct interactions (number of contacts). Direct interactions were defined as a distance between a pair of males less than 1.5 cm (the average size of a male), indicating they were touching. Additionally, we performed frame‐by‐frame analysis of the first 15 min of each video after the initial male landing to measure the duration of direct interactions when males came in contact with one another (contact duration, s).

To analyse males' behaviour on the LED, we counted the number of males that reached the LED in a time window of 30–37 min after the first male landed (number of males reaching the LED). For each male that reached the LED, we extracted the time they spent on the LED (time on LED, s), and the means by which they left the LED, classifying their exit as: (i) ‘Flight/walk’, indicating a voluntary departure from the LED; (ii) ‘Fall’, when a male fell from the LED alone; (iii) ‘Fall in aggregate’, when two or more males fell as a group simultaneously.

### 2.7 Statistical Analysis

All statistical analyses were conducted in R 4.3.2 (R Core Team 2023). All statistical tests used were from the base {stats} package unless otherwise stated.

To compare counts of males flying and walking to a dummy female in the arena, we used an Exact Binomial test against a probability of *p* = 0.5 that would be produced by males randomly selecting between these two movement strategies. For the comparison of the distances walked versus those flown by males in the arena, we used a Wilcoxon Rank Sign test against a null hypothesis of equality of the median (equal distances covered by both movement strategies). Likewise, the time taken by males to reach the pillar by flying versus those males that leaped or climbed down from the platform was compared using a Wilcoxon Rank Sign test against a null hypothesis of equality of the median (equal times taken by both movement strategies).

For field video recordings with the LED mounted on a trap, the statistical significance of the proportion of males reaching the trap was assessed using a one‐sample proportion test against a probability of *p* = 0.5 (equal probability of reaching/not reaching the trap). The number of males that flew, walked or combined walking and flying to reach the LED were compared with a chi‐squared test for count data against a probability of *p* = 1/3 that reflects a random selection of the three movement strategies. This was followed by post hoc pairwise binomial tests from the {rstatix} package (Kassambra 2023), against a random distribution of probability *p* = 0.5 to assess the significance of the difference between each pair of movement strategies. The significance of these tests was adjusted with the False Discovery Rate (Benjamini and Hochberg 1995).

To determine whether the time taken by males to reach the LED when located within a trap was influenced by the landing time, the landing distance and the number of neighbours, a maximal linear model was fitted, and nonsignificant terms were removed stepwise until only significant parameters remained (Table 1). Term significance was assessed using *F*‐tests and verified by selecting the model with the lowest AIC (Table 1). Nonsignificant terms were fitted post hoc with Spearman's correlations to illustrate the lack of relationship between time to LED, landing time and landing distance.

In field videos with the LED mounted on a wire, all generalised linear models were performed using the {glmmTMB} package (Brooks et al. 2017), and term significance was assessed using Z‐scores. The relationship between males' median distance to other males and the number of males on the ground was fitted with a generalised linear model with Gamma distribution and log‐link (Table 2). The relationship between the number of direct contacts and the number of males on the ground was fitted with a zero‐inflated Poisson generalised linear model with log‐link (Table 3). The relationship between the number of males landing and the number of males reaching the LED was fitted with a linear model.

A paired Wilcoxon Signed Rank test was used to compare the time spent by a male on the LED alone or with other males. The null hypothesis assumes equality of medians. The relationship between the time spent on the LED and the number of males reaching the LED was fitted with a negative binomial with log‐link (Table 4). The same model was also fitted excluding one apparent outlier datapoint (time on LED = 700 s), and no difference was found in comparison to the full data set. The different modes of exit from the LED were compared using a chi‐square test for probability against a random distribution of probability *p* = 1/3, followed by post hoc pairwise chi‐square tests against a random distribution of probability *p* = 0.5, with *p* values adjusted with False Discovery Rate (Benjamini and Hochberg 1995).

## 3 Results

### 3.1 Freely Moving Male Glow‐Worms in an Arena Combine Flight and Walking to Reach the Dummy Female

**FIGURE 3:**
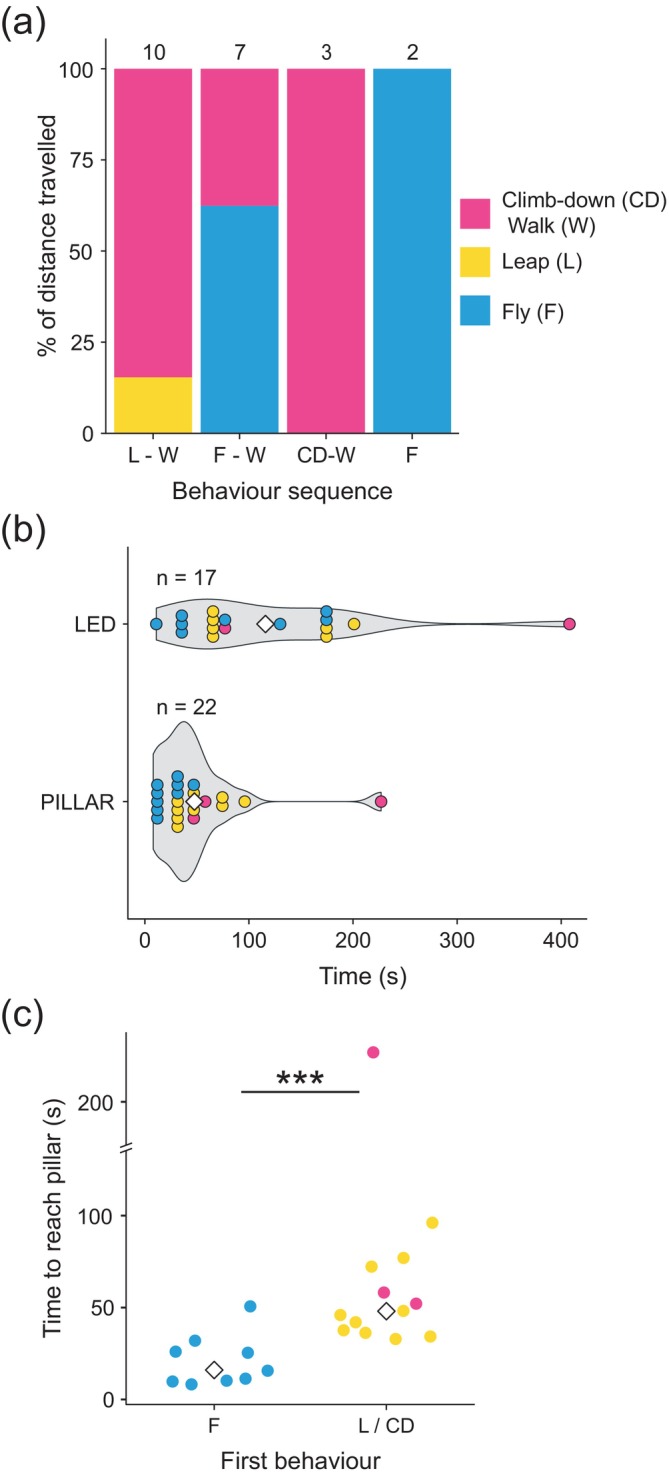
Freely moving males in an arena fly and walk to reach females. (a) The average percentage of the distance to the pillar travelled by male glow‐worms during each behaviour within the four observed behavioural sequences displayed as a stacked bar plot. Numbers at the top indicate the total number of males in each category. (b) Violin plots showing males' latency to reach either the pillar or the green LED. Dot colours correspond to those in (a). White diamonds indicate the mean of each group. (c) Males that climbed down or leaped from the platform took significantly longer to reach the pillar than those that flew. Dot colours correspond to those in (c). White diamonds represent the mean of each group. Significance (***, *p* < 0.001) is that of a Wilcoxon rank sum test comparing the time to reach the pillar. The single outlier does not affect the significance.

To determine whether male glow‐worms primarily fly or walk during the final stages of their approach to females, we recorded individual male paths towards a dummy female in an arena (see Section 2). Of 22 males placed individually in the arena (Figure 1), 10 leaped from the platform, whilst nine flew and three climbed down from the platform (Figure 3a). Two males flew directly to the pillar that held the dummy female, the remaining 20 walking at least part of the way (exact binomial test; flew vs. walked; *p* < 0.001, *N* = 22) (Figure 3a). This shows that most males walk to reach the dummy female over the short distances within the arena.

We quantified the time taken by males to reach the dummy female to determine which strategy (flying or walking) was faster. Five males reached the base of the pillar and 17 climbed to the dummy female. Of those males reaching the pillar, the majority did so in less than a minute (time to pillar; 48 ± 10 s; mean ± standard deviation) (Figure 3b). Of the males that reached the dummy female, two landed directly upon it, four males climbed the pillar directly, the remaining males (*N* = 11) walking around the pillar before climbing to the dummy female (time to LED; 116 ± 24 s) (Figure 3b). Overall, there was no significant difference in the distance males flew or walked (Wilcoxon rank sum test; flew vs. walked; *W* = 102.5, *p* = 0.7, *N* = 22). Males walked at 1 ± 0.3 cm s^−1^ (mean ± standard deviation) while they flew at 7 ± 4 cm s^−1^. Males that flew initially reached the pillar base significantly faster than those that leaped or climbed down from the platform (Wilcoxon rank sum test; flew vs. leaped‐climbed‐down/walked; *W* = 7, *p* < 0.001, *N* = 22; Figure 3c). Thus, although flight allows male glow‐worms to travel faster, most males walk to reach females over short distances.

### 3.2 In the Field, Male Glow‐Worms Land in the Vicinity of a Dummy Female Before Walking Towards It

**FIGURE 4:**
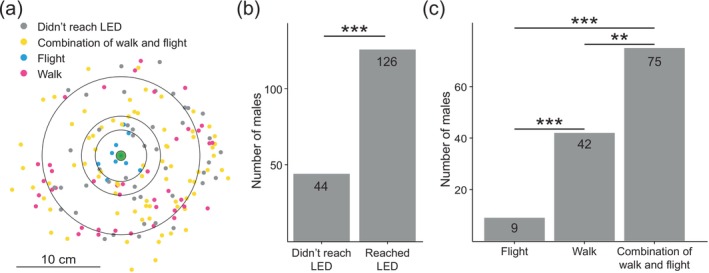
Males fly and walk to reach females in the wild. (a) Spatial locations of male landing positions on and around the dummy female (green LED). Grey dots represent individual male landing positions. (b) Count of males that reached the LED or did not. Significance (***, *p* < 0.001) is that of a one‐sample proportion test between the two groups. (c) Count of males that reached the LED using flight, walk or a combination of walking and flying. Significances (***, *p* < 0.001; **, *p* < 0.01) are those of pairwise binomial tests between each pair, adjusted with False Discovery Rate (see Section 2).

To determine which strategy, walking or flying, males use to reach a female in the field, we recorded 170 males approaching a dummy female trap (Figure 2c,d; see Section 2). Only 10 males landed within 5 cm of the dummy female (LED trap; see Section 2); seven (4.12%) on the rim of the trap and just three (1.76%) entering the trap directly (Figure 4a). The remaining ~94.12% landed near the trap (Figure 4a; 46.47% 5‐10 cm, 31.18% 10‐20 cm and 16.47% > 20 cm).

In total, 126 (74.1%) reached the dummy female, whereas 44 (25.9%) remained in or left the near vicinity of the trap. There was a significant difference between the proportion of males reaching the dummy female and those that did not (One‐sample proportion test; *X* ^2^ = 38.6, df = 1, *p* < 0.001, *N* = 170; Figure 4b). Thus, the dummy female trap is effective in attracting the majority of males, though a minority never reaches it.

Of the 126 males that reached the trap, 117 (92.9%) males walked at least part of the way towards the dummy female; 42 (33.3%) walked the entire way, and 75 (59.5%) combined walking with short flights to approach the dummy female, both greater than the 9 (7.1%) of males that flew all the way to the top of the trap or the dummy female (Figure 4c). There was a significant difference among the numbers of males adopting these three movement strategies (chi‐squared test; *X* ^2^ = 51.86, df = 2, *p* < 0.001, *N* = 126). There were also significant differences between each pair of movement strategies (pairwise binomial tests; walked/flew vs. walked, *p* < 0.01; walked vs. flew, *p* < 0.001; walked/flew vs. flew, *p* < 0.001; Figure 4c). Thus, in the field, few males reach dummy females in traps using flight exclusively, most males combining walking with flying instead.

### 3.3 The Time Taken by Males to Reach Females Is Independent of Their Landing Time or Landing Distance

**FIGURE 5:**
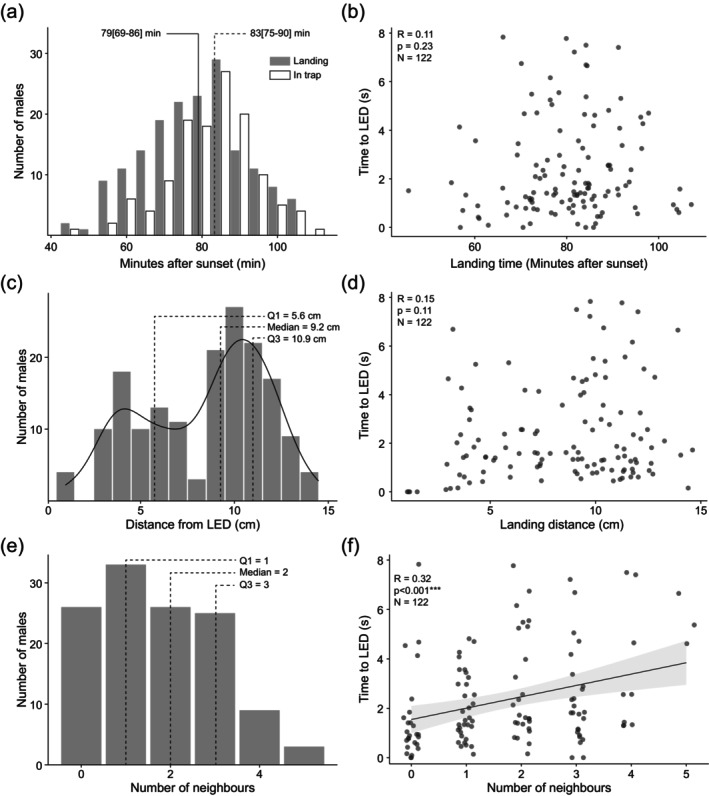
The presence of other males but not male landing time or landing distance influences the time taken to reach the dummy female. (a) Distribution of males' landing time (grey bars) and time at which they were caught in the LED (dummy female) trap (white bars). Lines indicate the median landing time (solid line) and median time at which they entered the trap (dotted line). (b) Time of landing and the time taken by males to reach the LED. (c) Distribution of males' landing distance from the LED. Dotted lines indicate from left to right the 25th percentile (Q1), median and 75th percentile (Q3). The black line is a smoothed representation of the count data. (d) Landing distance from and the time taken by males to reach the LED. (e) Distribution of the number of neighbours. Dotted lines indicate from left to right the 25th percentile (Q1), median and 75th percentile (Q3). (f) Time taken by a male to reach the LED was dependent upon the number of males present in the area. The significant linear regression and 95% confidence interval are shown by a grey line and surrounding pale grey region, respectively. Dark grey dots represent individual males (see Table 1 for model significance).

To determine how landing time and landing distance influence the time males take to reach females, we measured the landing time of individual males and the time they take to reach the dummy female. Males landed 79 [69–86] min (median [Q1–Q3]) after sunset and entered the trap 83 [75–90] min after sunset (Figure 5a). Consequently, males took 1.6 [0.9–3.3] min to reach the dummy female. The time taken by males to reach the dummy female was independent of their landing time (Spearman's correlation; *R* = 0.11, *p* = 0.23, *N* = 122; Figure 5b). Males landed 9.22 [5.68–10.93] cm (median [Q1–Q3]) from the LED (Figure 5c) and walked most of the distance to reach the dummy female. The time taken by males to reach the dummy female was independent of their landing distance (Spearman's correlation; *R* = 0.15, *p* = 0.11, *N* = 122; Figure 5d). This suggests that arriving earlier or landing nearer did not enable them to reach the dummy female sooner.

### 3.4 Males Are Slowed by the Presence of Other Males Near the Trap

To determine whether the presence of other males in the vicinity influences the time males take to reach females, we measured the number of other males present (neighbours) when a male landed. The number of neighbours ranged from 0 to 5 (2 [1–3]; median [Q1–Q3]; Figure 5e). The time taken to reach the dummy female was correlated with the number of neighbours (Linear regression; *R* = 0.32, *p* < 0.001, *N* = 122; Figure 5f). Indeed, the number of neighbours significantly predicted the time taken to reach the dummy female in a linear model when landing time and landing distance were also included (Table 1). This suggests that males' progress towards the dummy female is slowed by the presence of other males in the vicinity.

**TABLE 1:** Summary of the maximal linear model and sequential model simplification used to obtain the minimum adequate model describing the relationship between time to LED and the different landing parameters (distance, time and number of neighbours).

	Terms	*F*	*p*	AIC
*Maximal model*				
Time to LED ~		**5.4**	< 0.01**	**156**
	Number of neighbours	11.6	< 0.001***	165
	Landing time	0.01	0.9	154
	Landing distance	3.3	0.07	157
*Intermediate model*				
Time to LED ~		**6.4**	< 0.01**	**157**
	Number of neighbours	12.2	< 0.001***	167
	Landing time	0.001	0.9	155
*Minimal model*				
Time to LED ~		**12.9**	< 0.001***	**155**
	Number of neighbours	12.9	< 0.001***	166

*Note:* *F*‐value, significance and AIC are given for the fitted model in bold, and for each single term deletion (nonbold).

### 3.5 Males Interact on the Ground Near the Dummy Female LED

**FIGURE 6:**
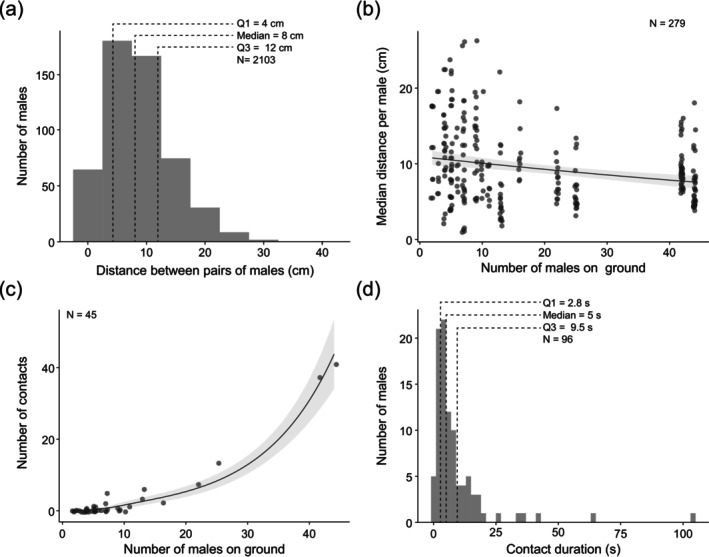
Glow‐worm males interact on the ground near the dummy female. (a) Distribution of the distance between pairs of males found on the ground around the wire‐mounted dummy female LED. (b) The median distance between males correlates with the number of males present on the ground. Model prediction and 95% confidence intervals are shown as a black line and surrounding grey region. Dots represent the number of males on the ground visible per frame (see Table 2 for model significance). (c) The number of direct contacts between males increases with the number of males on the ground. Model prediction and 95% confidence intervals are shown as a black line and surrounding grey region. Dots represent the number of contacts in each frame (see Table 3 for model significance). (d) Distribution of the duration of direct contacts between one or more males.

We assessed the nature of possible interactions between males in the field by recording 424 males landing nearby or on the dummy female LED (Figure 2e,f; see Section 2) measuring 2103 distances between pairs of males. The median distance between any pair of males after landing was 8 [4–12] cm (Figure 6a). This distance decreased significantly with the number of males on the ground at that time (Generalised linear model (gamma); Table 2, Figure 6b). The number of direct contacts between males increased with the number of males present on the ground (Figure 6c). This increase was significant and became more pronounced with greater numbers of males present on the ground (Generalised linear model (zero‐inflated Poisson); Table 3, Figure 6c). To determine the duration of direct contacts between males, we observed a total of 96 direct interactions between males (see Section 2). Although some contacts lasted longer than 25 s, most contacts lasted for 5 [2.8–9.5] seconds (Figure 6d). Thus, once landed, males are in proximity and frequently engage in direct though typically brief interactions.

**TABLE 2:** Summary of the generalised linear model (Gamma family) used to describe the relationship between the median distance between males and the number of males present on the ground.

Formula	Estimate	Std. error	*Z*	*p*
Median distance per male ~	2.4	0.05	50	< 0.001***
Number of males on the ground	**−0.01**	**0.002**	**−4.4**	< 0.001***

*Note:* Estimate, standard error, *Z*‐value and significance are given for the intercept and model term. Significant terms are shown in bold.

**TABLE 3:** Summary of the zero‐inflated generalised linear model (Poisson family) used to describe the relationship between the number of direct contacts between males and the number of males present on the ground.

Formula	Estimate	Std. error	*Z*	*p*
*Conditional model*				
Number of contacts ~	−0.06	0.27	−0.22	0.826
Number of males on the ground	**0.09**	**0.007**	**12.12**	< 0.001***
*Zero‐inflation model*				
Number of contacts ~	3.3	1.18	2.8	< 0.01**
Number of males on the ground	**−0.4**	**0.18**	−**2.4**	< 0.05*

*Note:* Estimate, standard error, Z‐value and significance are given for the intercept and model term of the conditional and zero‐inflated models. Significant terms are shown in bold.

### 3.6 Greater Numbers of Males Reduce the Likelihood of Reaching the Dummy Female

**FIGURE 7:**
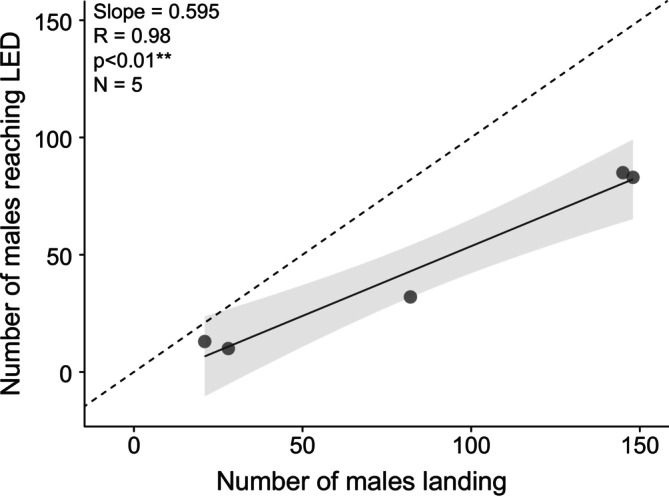
The number of males landing impacts the number of males successfully reaching the dummy female. Number of males reaching the LED depends on the number of males that landed. The significant linear regression and 95% confidence interval are shown by a grey line and surrounding pale grey region, respectively. Dark grey dots represent individual nights. The dotted line illustrates the identity line.

To determine the effect of male–male interactions on their ability to reach the female, we recorded a total of 223 males reaching the dummy female (Figure 2e,f; see Section 2). The greater the number of males landing in the vicinity of the dummy female, the greater the number of males that reached it (Linear regression; *R* = 0.98, *p* < 0.001, *N* = 5; Figure 7). However, as greater numbers of males landed, the rate at which they reached the dummy female decreased (Linear regression; slope = 0.595; Figure 7). This suggests that the number of males present affects their ability to reach the dummy female.

### 3.7 Male–Male Interactions Influence the Time Spent on the Dummy Female

**FIGURE 8:**
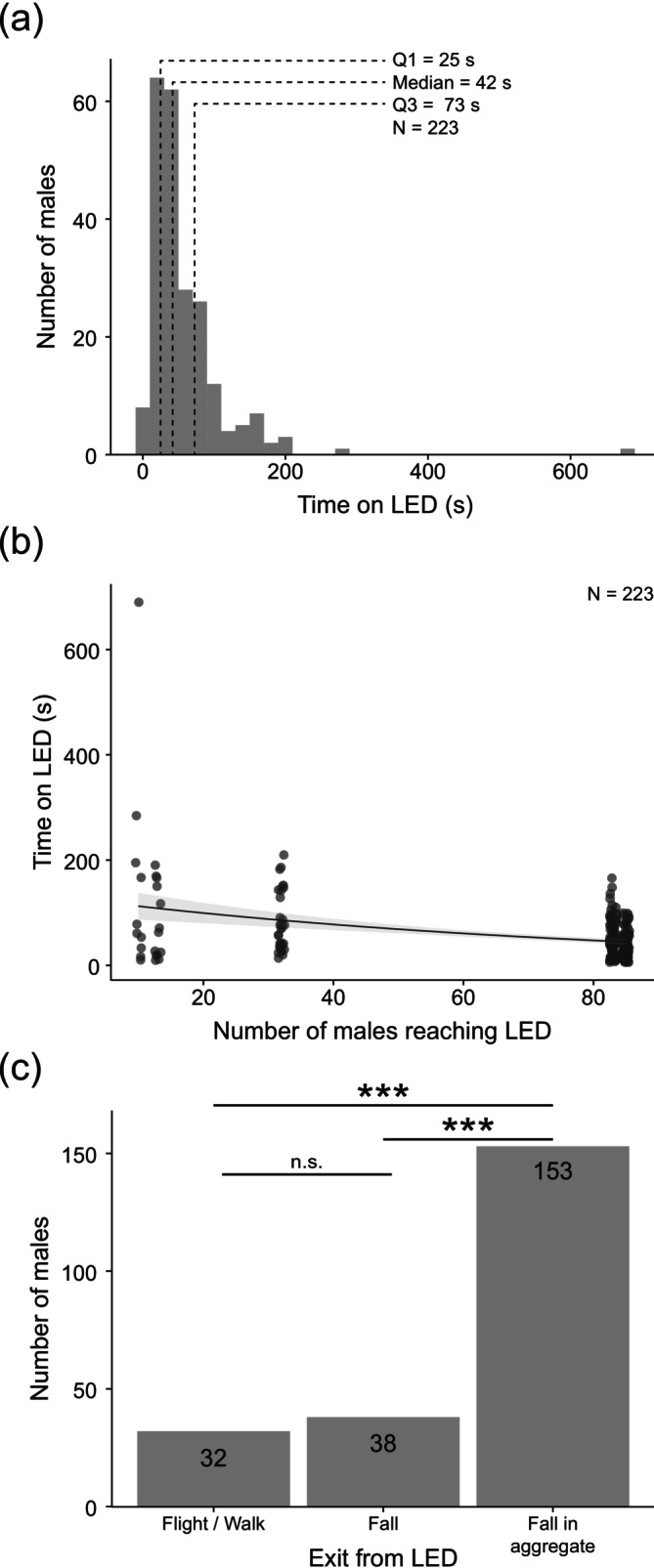
Male–male interactions influence reaching and remaining on the dummy female. (a) Distribution of the time each male spent on the LED. Dotted lines indicate from left to right the 25th percentile (Q1), median and 75th percentile (Q3). (b) The time spent by males on the LED depending on the number of males that reached the LED. Model prediction and 95% confidence intervals are shown as a black line and surrounding grey region. Dots represent the number of contacts in each individual frame (see Table 4 for model significance). (c) Count of males that left from the LED by choice (flight/walk) or by falling either individually (fall) or in a group (fall in aggregate). Significances (***, *p* < 0.001) are that of pairwise chi‐square tests between all groups, adjusted with False Discovery Rate.

To determine the effect of male–male interactions once they have reached the female, we recorded male behaviour on the dummy female. Between 1 and 11 males (2 [1–3]) were present on the dummy female at any given time. During that time, males were in direct contact with one another, frequently climbing onto or walking under one another. Males spent, on average, 42 [25–73] seconds on the LED (Figure 8a). Males spent 33 [16–59] seconds on the LED in the presence of other males, significantly longer than when alone on the LED (0 [0–9] s) (Wilcoxon paired signed rank test; *V* = 4173, *p* < 0.001, *N* = 223). The time each male spent on the dummy female decreased with greater numbers of males reaching it (Generalised linear model (Negative binomial); Table 4, Figure 8b). Just 14% of males voluntarily left the LED by flying or walking, significantly different from the 86% who fell from the LED (chi‐squared test; *X* ^2^ = 125.12, df = 2, *p* < 0.001, *N* = 223; Figure 8c). Of the males that fell from the LED, a significant majority (80%) fell in aggregates 2–8 rather than alone (Pairwise chi‐squared tests; Fall versus Fall in aggregate, *p* < 0.001; Figure 8c), and significantly more commonly than the number of males leaving voluntarily (flight/walk vs. fall in aggregate, *p* < 0.001; Figure 8c). A similar number of males fell on their own and left voluntarily (pairwise chi‐squared tests; flight/walk vs. fall, *p* = 0.47; Figure 8c). This indicates that glow‐worm males engage in direct male–male contacts on the LED that affect the time they spend attempting to mate with the female.

**TABLE 4:** Summary of the generalised linear model (negative binomial family) used to describe the relationship between the time spent by each male on the LED and the number of males that reached the LED.

Formula	Estimate	Std. error	*Z*	*p*
Time on LED ~	4.8	0.13	37	< 0.001***
Number of males reaching LED	**−0.01**	**0.002**	**−6.9**	< 0.001***

*Note:* Estimate, standard error, *Z*‐value and significance are given for the intercept and model term. Significant terms are shown in bold.

## 4 Discussion

Our aim was to understand how male glow‐worms approach glowing females during the final stages of their scramble in the dark. To achieve this, we combined observations from a behavioural arena and in the field with detailed quantitative analysis of males' movements. Our analysis of individual male behaviour within a laboratory arena showed that they mainly walk part or all of their approach to dummy females over these short distances. To determine whether this was indicative of male behaviour in natural environments, we used infrared videography in the field. Again, this showed that most male glow‐worms land in the near vicinity of dummy females and walk part or all of the final stage of their approach, with only a small minority reaching the dummy female exclusively by flying. This contrasts with previous descriptions of glow‐worm mating that suggest that most males fly directly to the female (Tyler 2002). We found that the time taken by males to reach dummy females after their initial landing depended upon the number of other males present. Further videography revealed that male glow‐worms engage in frequent, often brief interactions that delay their progress to the dummy female and may prevent them from reaching the dummy female entirely. These interactions continue whilst they are on the dummy female and are sufficient to cause males to fall, preventing them from continuing their courtship. This suggests that for most male glow‐worms, the final stages of the scramble for females after their initial landing are influenced by male–male interactions that may be costly in terms of lost opportunities for reproduction.

Our field videos show that more than 20 males can land near a single female within a short period, with up to six approaching the female simultaneously, consistent with a skewed operational sex ratio with males typically outnumbering females at sites at which they are present (AJAS and JEN, unpublished observation). Together with our observations of male movement during the final stages of approach to the female, this suggests a scenario in which males fly across the landscape in prolonged searches to detect a female glow, after which they land close to the female and continue their scramble on foot or using short, bounding flights; few males fly directly to the female. When combined with previously documented aspects of glow‐worm life history, this suggests that their mating system (reviewed in Section 1) could be described as prolonged searching polygyny, with males engaged in scramble competition (Emlen and Oring 1977; Thornhill and Alcock 1983; Herberstein et al. 2017).

The mating strategy of male glow‐worms is reminiscent of that of other male insects engaged in scramble competition to reach sedentary females that advertise their position. For example, only 17% of male potato tuber moths (*Phthorimaea operculella*) reach a dummy female (pheromone source) suspended from the ceiling through flight, while 68% of males can reach a dummy female sitting on a wall by walking (Ono 1985). Male moths land 5–65 cm away from the dummy female and walk the remainder of the distance (Ono 1985). Yet it is also like that of some parasitoids searching for prey. For example, female parasitoid flies ( *Ormia ochracea* ) locate male crickets advertising their position to female crickets using their song (Müller and Robert 2001). The female fly uses phonotaxis to find the male cricket, after which they deposit their larvae. Female *Ormia* land ~8.2 cm from a dummy cricket before walking the remaining distance, comparable to our observation of male glow‐worms landing near a dummy female in our videos. This suggests that combining flight over longer distances with walking over short distances may be a common strategy in insects for approaching small stationary targets that are advertising their position: walking permits fine‐tuning of insects' goal‐directed orientation in complex habitats, such as dense vegetation.

Yet some aspects of male glow‐worms' approach to females do not seem to conform to typical aspects of scramble competition. For some insect species (reviewed in Herberstein et al. 2017), including fireflies (Vencl and Carlson 1998; Vencl 2004), directed and rapid locomotion is a feature of scramble competition among males and is a major determinant of mating success. Yet male glow‐worms approaching a dummy female that arrived earlier or landed nearer did not necessarily reach it faster. Several factors may explain this disparity. The time of arrival in the near vicinity of a female (or dummy female) likely depends to some extent on the distances males need to fly as well as their flight speed. Although the distance they must fly will depend on their previous landing position and will therefore be dependent upon female dispersal across the landscape, flight speed is likely dependent upon male size, with larger males flying further faster and arriving at females earlier. Indeed, in male fireflies ( *Photinus pyralis* ), larger wings are favoured to facilitate patrol flights to find sedentary females (Vencl and Carlson 1998; Vencl 2004). Yet, once on the ground, smaller males may be more manoeuvrable and better able to engage in short, bounding flights that our data suggest cover distance more rapidly. This appears to be the case in several other insect species (e.g., Alcock et al. 2010), including fireflies (Vencl and Carlson 1998). Due to the resolution of the infrared video we obtained and the distortion of the field of view, we were unable to accurately determine the lengths of individual male glow‐worms, which may have provided further insight into the variation in the sizes of male glow‐worms reaching the dummy female.

A further factor that may influence male glow‐worms' movement towards the female post‐landing is the occlusion of the glow by males that have already reached the female. Our data suggest that only a small proportion of males (~11%) contacted the dummy female (LED) when it was located within a trap, and these males remained there only briefly. Moreover, given the size of the LED, it is unlikely that a single male glow‐worm could occlude the signal entirely. Thus, it is unlikely that occlusion of the dummy female signal is the cause of the absence of a relationship between arrival times or landing distances and reaching the female in our data. However, a higher proportion of males (~53%) contacted the dummy female when it was exposed on a wire. Males remained on the LED for relatively short periods, typically less than 1 min, but often formed clusters around the LED that could have occluded substantial portions of the signal, which may contribute to a relative reduction in the rate at which males reach the dummy female as their numbers increase. The signal produced by a female glow‐worm is emitted from a smaller region on the abdomen (Tyler 2002) and could be more easily occluded. Were similar numbers of males able to reach the female glow‐worm, signal occlusion could play an important role in reducing male glow‐worm attraction to females at which other males are already present.

The time taken for males to reach the dummy female (LED) in a trap increased with increasing numbers of other males present at the time of arrival. One explanation for this is that males may impede one another after they have landed and are locomoting towards a female. This is supported by our analysis of males approaching the dummy female attached to a wire. In this scenario, males land and aggregate in the vicinity of the dummy female. Such aggregation produces male–male interactions that increase with increasing numbers of males. Although each interaction is brief, typically lasting 5 s (though they can be considerably longer), accumulated interactions may slow the males in their approach to the dummy female. Moreover, as males and the interactions between males increase, the rate at which males reach females declines, suggesting that interactions are a substantial impediment to males. These interactions occur on the ground rather than on the dummy female; however, clusters of up to 11 males also gather on the dummy female. Males in these clusters engage in frequent interactions and often fall from the dummy female together. This suggests that male–male interactions are sufficient to curtail the time males have engaging with the dummy female.

**FIGURE 9:**
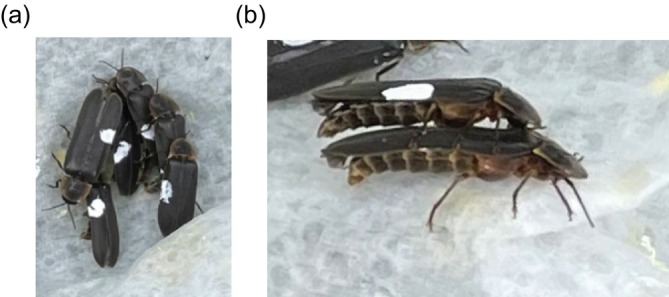
Males can impede each other's movements. (a) Photograph of over eight males aggregating after being placed in an enclosed space. (b) Photograph showing a male mounted on top of another.

Similar behaviour has been documented in *Photinus pyralis* fireflies (Vencl and Carlson 1998). In these fireflies, males aggregate in large numbers and engage in intense scrambles with males clambering over one another, described by Vencl and Carlson (1998) as ‘love‐knots’. They demonstrated this behaviour in males captured on the previous evening and released in aggregate in glass tubes in the absence of females, whereas our observations are in the presence of males that have accumulated in the field in the presence of a dummy female. Male glow‐worms placed in confined spaces also aggregate and form ‘knots’ (Figure 9a) in which males are often observed mounted on others (Figure 9b). Thus, love‐knots form when males are in proximity and contribute to both the reduction in the rate of reaching the dummy female and the greater time taken when they do.

Taken together, our findings allow us to propose a model for the final stages of male glow‐worm scramble towards females, though it is important to recognise that this is derived from experiments that employ dummy females in open areas rather than female glow‐worms in dense vegetation. We propose that male glow‐worms scour the landscape to detect signalling females for mating. Once males detect a female, most land nearby, thereafter walking or making short, bounding flights to cover the final distance. Few males fly directly to the female. During this final stage of approach, males encounter other males that have landed and are also attempting to reach the female. Males can interfere with the progress of other males, slowing them down or even preventing them from reaching the female. Once males reach the female, they continue to interact, and these interactions may dislodge them from the female, causing them to fall. These features of the later stages of the males' scramble to find females suggest that male–male interactions are frequent and impose a considerable cost in terms of delaying or even preventing males' access to females and reducing their opportunities for reproduction.

## Author Contributions

**Estelle M. Moubarak:** conceptualization (equal), data curation (lead), formal analysis (lead), investigation (lead), methodology (equal), project administration (supporting), resources (equal), software (lead), validation (equal), visualization (lead), writing – original draft (equal), writing – review and editing (equal). **Alan J. A. Stewart:** funding acquisition (equal), resources (equal), writing – review and editing (supporting). **Jeremy E. Niven:** conceptualization (equal), formal analysis (supporting), funding acquisition (equal), methodology (equal), project administration (lead), resources (equal), supervision (lead), validation (equal), visualization (supporting), writing – original draft (equal), writing – review and editing (equal).

## Ethics Statement

Approval given by Sussex Animal Welfare and Ethical Review Body (reference ARG‐34‐JN1 for field work and ARG‐34‐JN2 for behavioural laboratory work).

## Conflicts of Interest

The authors declare no conflicts of interest.

## Data Availability

All data files associated with this study are available online (https://doi.org/10.5061/dryad.83bk3jb4k).

## References

[ece372187-bib-0001] Alcock, J. 1997. “Small Males Emerge Earlier Than Large Males in Dawson's Burrowing Bee ( *Amegilla dawsoni* ) (Hymenoptera: Anthophorini).” Journal of Zoology 242, no. 3: 453–462. 10.1111/j.1469-7998.1997.tb03848.x.

[ece372187-bib-0002] Alcock, J. , W. J. Bailey , and L. W. Simmons . 2010. “The Mating System of *Amegilla (Asarapoda) paracalva Brooks* (Hymenoptera: Apidae).” Journal of Insect Behaviour 23, no. 1: 69–79. 10.1007/s10905-009-9196-x.

[ece372187-bib-0003] Bateman, A. J. 1948. “Intra‐Sexual Selection in *Drosophila* .” Heredity 2, no. 3: 349–368. 10.1038/hdy.1948.21. 18103134

[ece372187-bib-0004] Baudry, G. , J. Hopkins , P. C. Watts , and A. Kaitala . 2021. “Female Sexual Signaling in a Capital Breeder, the European Glow‐Worm *Lampyris noctiluca* .” Journal of Insect Behaviour 34, no. 1–2: 16–25. 10.1007/s10905-020-09763-9.

[ece372187-bib-0005] Benjamini, Y. , and Y. Hochberg . 1995. “Controlling the False Discovery Rate: A Practical and Powerful Approach to Multiple Testing.” Journal of the Royal Statistical Society: Series B: Methodological 57, no. 1: 289–300. 10.1111/j.2517-6161.1995.tb02031.x.

[ece372187-bib-0006] Bird, S. , and J. Parker . 2014. “Low Levels of Light Pollution May Block the Ability of Male Glow‐Worms ( *Lampyris noctiluca* L.) to Locate Females.” Journal of Insect Conservation 18, no. 4: 737–743. 10.1007/s10841-014-9664-2.

[ece372187-bib-0007] Brooks, M. E. , K. Kristensen , K. J. van Benthem , et al. 2017. “glmmTMB Balances Speed and Flexibility Among Packages for Zero‐Inflated Generalized Linear Mixed Modeling.” R Journal 9, no. 2: 378–400. 10.32614/rj-2017-066.

[ece372187-bib-0008] Emlen, S. T. , and L. W. Oring . 1977. “Ecology, Sexual Selection, and the Evolution of Mating Systems.” Science 197, no. 4300: 215–223. 10.1126/science.327542. 327542

[ece372187-bib-0009] Herberstein, M. E. , C. J. Painting , and G. I. Holwell . 2017. “Scramble Competition Polygyny in Terrestrial Arthropods.” In Advances in the Study of Behaviour, 237–295. Elsevier Ltd. 10.1016/bs.asb.2017.01.001.

[ece372187-bib-0010] Hopkins, J. , T. K. Lehtonen , G. Baudry , and A. Kaitala . 2021. “Costly Mating Delays Drive Female Ornamentation in a Capital Breeder.” Ecology and Evolution 11, no. 13: 8863–8868. 10.1002/ece3.7719.

[ece372187-bib-0011] Ineichen, S. , and B. Rüttimann . 2012. “Impact of Artificial Light on the Distribution of the Common European Glow‐Worm, *Lampyris noctiluca* (Coleoptera: Lampyridae).” Lampyrid 2: 31–36.

[ece372187-bib-0012] Kassambra, A. 2023. “Package “rstatix”: Pipe‐Friendly Framework for Basic Statistical Tests.” 0.7.2, 100. https://rpkgs.datanovia.com/rstatix/.

[ece372187-bib-0013] Kelly, C. D. , L. F. Bussière , and D. T. Gwynne . 2008. “Sexual Selection for Male Mobility in a Giant Insect With Female‐Biased Size Dimorphism.” American Naturalist 172, no. 3: 417–423. 10.1086/589894. 18651830

[ece372187-bib-0014] Kivelä, L. , C. Elgert , T. K. Lehtonen , and U. Candolin . 2023. “The Color of Artificial Light Affects Mate Attraction in the Common Glow‐Worm.” Science of the Total Environment 857: 159451. 10.1016/j.scitotenv.2022.159451. 36252663

[ece372187-bib-0015] Kvarnemo, C. , and I. Ahnesjo . 1996. “The Dynamics of Operational Sex Ratios and Competition for Mates.” Trends in Ecology & Evolution 11, no. 10: 404–408. 10.1016/0169-5347(96)10056-2. 21237898

[ece372187-bib-0016] Lewis, S. M. , and C. K. Cratsley . 2008. “Flash Signal Evolution, Mate Choice, and Predation in Fireflies.” Annual Review of Entomology 53, no. 1: 293–321. 10.1146/annurev.ento.53.103106.093346. 17877452

[ece372187-bib-0017] Moubarak, E. M. , A. S. D. Fernandes , A. J. A. Stewart , and J. E. Niven . 2023. “Artificial Light Impairs Local Attraction to Females in Male Glow‐Worms.” Journal of Experimental Biology 226, no. 11: jeb245760. 10.1242/jeb.245760. 37311409 PMC10281516

[ece372187-bib-0018] Müller, P. , and D. Robert . 2001. “A Shot in the Dark: The Silent Quest of a Free‐Flying Phonotactic Fly.” Journal of Experimental Biology 204, no. 6: 1039–1052. 10.1242/jeb.204.6.1039. 11222123

[ece372187-bib-0019] Myers, S. S. , T. R. Buckley , and G. I. Holwell . 2015. “Mate Detection and Seasonal Variation in Stick Insect Mating Behaviour (Phamatodea: *Clitarchus hookeri*).” Behaviour 152, no. 10: 1325–1348. 10.1163/1568539X-00003281.

[ece372187-bib-0020] Ono, T. 1985. “Male Approach to the Female and the Role of Two Pheromone Components in the Potato Tuber Moth, *Phthorimaea operculella* (Lepidoptera: Gelechiidae).” Applied Entomology and Zoology 20, no. 1: 34–42. 10.1303/aez.20.34.

[ece372187-bib-0021] R Core Team . 2023. R: A Language and Environment for Statistical Computing. R Foundation for Statistical Computing. https://www.r‐project.org/.

[ece372187-bib-0022] Schindelin, J. , I. Arganda‐Carreras , E. Frise , et al. 2012. “Fiji: An Open‐Source Platform for Biological‐Image Analysis.” Nature Methods 9, no. 7: 676–682. 10.1038/nmeth.2019. 22743772 PMC3855844

[ece372187-bib-0023] Stewart, A. J. A. , C. D. Perl , and J. E. Niven . 2020. “Artificial Lighting Impairs Mate Attraction in a Nocturnal Capital Breeder.” Journal of Experimental Biology 223, no. 19: 1–5. 10.1242/jeb.229146. 32665443

[ece372187-bib-0024] Thornhill, R. , and J. Alcock . 1983. The Evolution of Insect Mating Systems. Harvard University Press. 10.4159/harvard.9780674433960.

[ece372187-bib-0025] Tyler, J. 2002. The Glow‐Worm. Lakeside P. Kent.

[ece372187-bib-0026] Van den Broeck, M. , R. De Cock , S. Van Dongen , and E. Matthysen . 2021. “Blinded by the Light: Artificial Light Lowers Mate Attraction Success in Female Glow‐Worms ( *lampyris noctiluca* L.).” Insects 12, no. 8: 734. 10.3390/insects12080734. 34442300 PMC8397135

[ece372187-bib-0027] Vencl, F. V. 2004. “Allometry and Proximate Mechanisms of Sexual Selection in *Photinus* Fireflies, and Some Other Beetles.” Integrative and Comparative Biology 44, no. 3: 242–249. 10.1093/icb/44.3.242. 21676703

[ece372187-bib-0028] Vencl, F. V. , and A. D. Carlson . 1998. “Proximate Mechanisms of Sexual Selection in the Firefly *Photinus pyralis* (Coleoptera: Lampyridae).” Journal of Insect Behaviour 11, no. 2: 191–207. 10.1023/A:1021091806472.

[ece372187-bib-0029] Wells, K. D. 1977. “The Social Behaviour of Anuran Amphibians.” Animal Behaviour 25, no. part 3: 666–693. 10.1016/0003-3472(77)90118-X.

[ece372187-bib-0030] Wiklund, T. , and C. Fagerström . 1982. “Why Do Males Emerge Before Females? Protandry as a Mating Strategy in Male and Female Butterflies.” Oecologia 52: 164–166. 28310501 10.1007/BF00363830

